# Pnictide-based colloidal quantum dots for infrared sensing applications

**DOI:** 10.1186/s40580-025-00489-y

**Published:** 2025-05-29

**Authors:** Jaeyoung Seo, Seongchan Kim, Dongjoon Yeo, Namyoung Gwak, Nuri Oh

**Affiliations:** Division of Materials Science and Engineering, Hanyang University, 222 Wangsimni-ro, Seongdong-gu, Seoul, 04763 Republic of Korea

**Keywords:** Colloidal quantum dots, III–V compound semiconductor, Infrared photodetector

## Abstract

Pnictide-based quantum dots (QDs) have emerged as promising materials for next-generation infrared photodetectors due to their superior physical and electrical properties. Among them, InAs and InSb QDs are particularly attractive for their tunable bandgaps in the short-wave infrared (SWIR) region, high carrier mobility, and compatibility with solution-based, large-area, and low-cost fabrication processes. This review discusses recent advancements in the synthesis of InAs and InSb QDs, focusing on precursor strategies and surface engineering techniques to enhance their optical and electronic properties. Additionally, we explore their integration into infrared photodetectors, analyzing current performance and limitations. Finally, we outline future research directions aimed at further enhancing material properties and device performance, paving the way for the broader adoption of III–V QDs in next-generation infrared technologies.

**Graphical Abstract:**

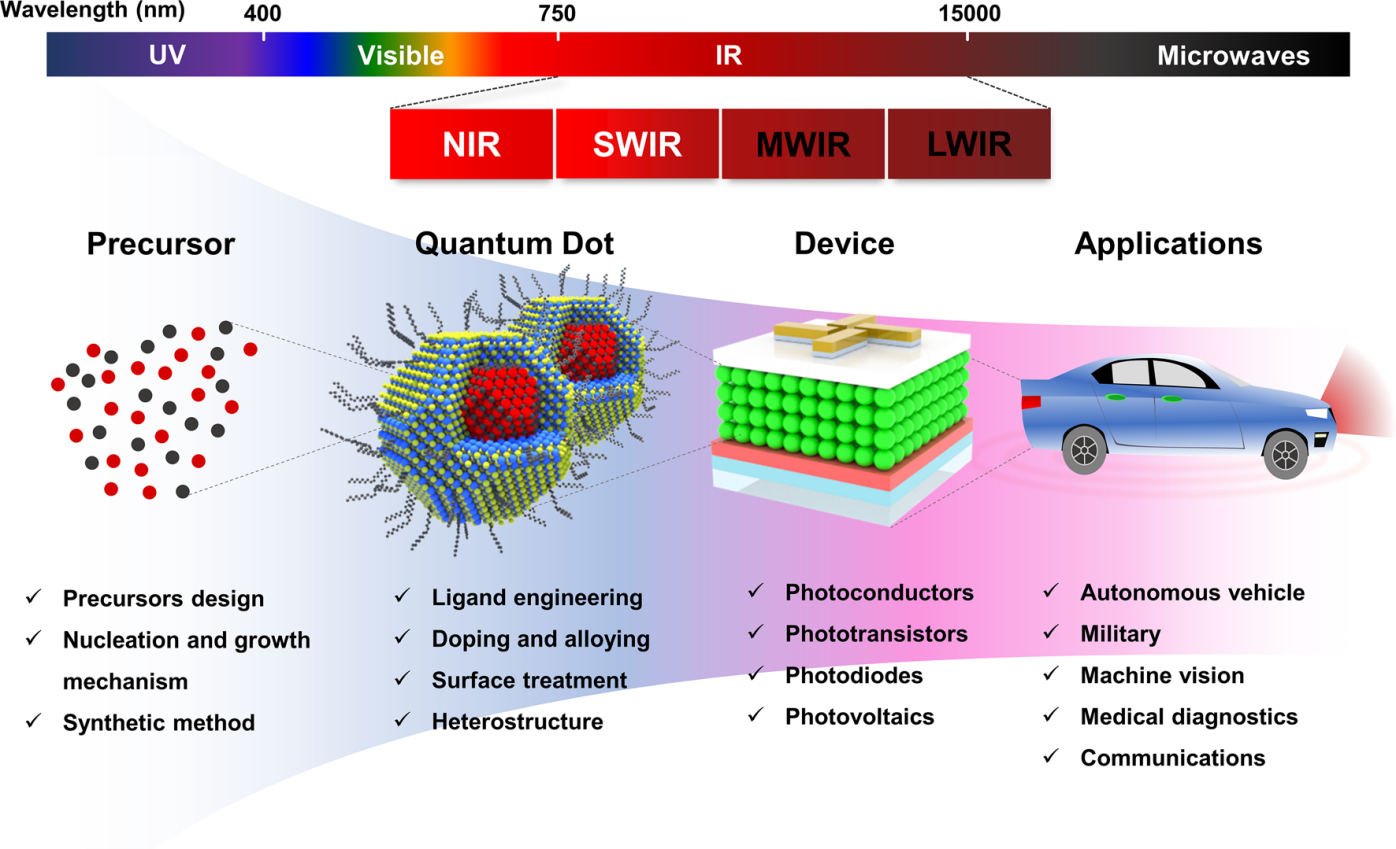

## Introduction

Photodetectors are indispensable in modern technology, enabling real-time monitoring of industrial processes, environmental conditions, and human activities. They play a critical role in a wide range of applications, including optical communication [1, 2], consumer electronics [3], solar energy harvesting [4], and medical diagnostics [5]. As the demand for high-performance photodetectors continues to grow, the development of novel materials and device architectures has become a key focus to enhance their sensitivity, stability, and operational efficiency. Among different types of photodetectors, short wavelength infrared (SWIR) photodetectors have gained significant attention for their unique operational advantages. Unlike mid-wave infrared and long-wave infrared photodetectors, which primarily image thermal radiation emitted from objects, SWIR photodetectors operate under active illumination conditions, detecting light that is reflected or transmitted by the target—analogous to visible light imaging systems. This feature allows SWIR photodetectors to deliver superior image contrast and high-resolution sensing, making them ideal for applications such as night vision, industrial inspection, and remote sensing [6]. Moreover, their spectral positioning allows SWIR photodetectors to minimize interference from ambient light sources, ensuring high accuracy in challenging environments. Furthermore, SWIR detection is critical in applications that require reduced human exposure to high-energy radiation [7]. 

Currently, the most commonly used materials for IR photodetectors include silicon (Si), germanium (Ge), and indium gallium arsenide (InGaAs). However, Si-based IR sensors exhibit limitations in SWIR detection due to their indirect bandgap (1.1 eV), which results in low sensitivity and poor photoelectric efficiency in this spectral range [8]. Although bulk materials such as InGaAs and Ge offer superior performance, their high fabrication costs remain a significant barrier to broad adoption [9]. Additionally, their intrinsic material properties hinder their integration into emerging device architectures that require flexibility, foldability, and miniaturization, thus limiting their potential in next-generation optoelectronic systems [10, 11]. 

Infrared colloidal quantum dots (QDs) have emerged as a compelling alternative to conventional bulk materials, effectively overcoming their inherent limitations. When the QD size is smaller than the exciton Bohr radius, the quantum confinement effect becomes significant, enabling precise bandgap tunability through size control [12–14]. Furthermore, QDs can be synthesized via low-temperature, wet-chemical methods, enabling cost-effective and large-area fabrication through solution-based processing [15, 16]. These advantages make QDs highly attractive for solution-processable optoelectronic applications.

Historically, IR QDs have predominantly been developed using heavy-metal-based materials, particularly chalcogenide QDs—IV–VI compounds such as PbS, PbSe, and PbTe, or II–VI compounds like HgS, HgSe, HgTe [17–22]. Building on this, a monolithic approach has been adopted to enable the direct fabrication of PbS QD photodiodes onto CMOS readout integrated circuits via solution-based processing [23]. However, despite their high IR sensitivity, the environmental toxicity of heavy-metal-based QDs, coupled with stringent regulations such as the “Restriction of Hazardous Substances” (RoHS), pose significant barriers to commercialization [24]. Compared to commercialized SWIR photodetectors such as InGaAs, colloidal QDs still face challenges including lower carrier mobilities, surface trap-induced recombination, and environmental instability, all of which can adversely affect device performance [25–27]. Nevertheless, QDs offer distinct advantages over conventional bulk materials, and ongoing advances in colloidal synthesis and surface engineering are rapidly closing this gap, suggesting strong potential for integration and broader application opportunities.

To address these challenges, growing research efforts have been directed toward developing heavy-metal-free QD materials. Among these, pnictide (II–V and III–V) QDs and I–VI QDs have gained significant attention. Ag_2_X (X = S, Se, Te) present a non-toxic alternative heavy-metal-based QDs; however, its performance in SWIR detection remains limited by several factors. Due to their relatively weak quantum confinement, I-VI QDs exhibit limited bandgap tunability and reduced excitonic character. These factors, combined with intrinsically low oscillator strength and broad electronic transitions, contribute to weak and poorly defined absorption features. Furthermore, their electrical and optical properties remain suboptimal due to the high mobility of monovalent silver ions and the presence of surface defects, both of which degrade device performance [28–31]. 

**Fig. 1:**
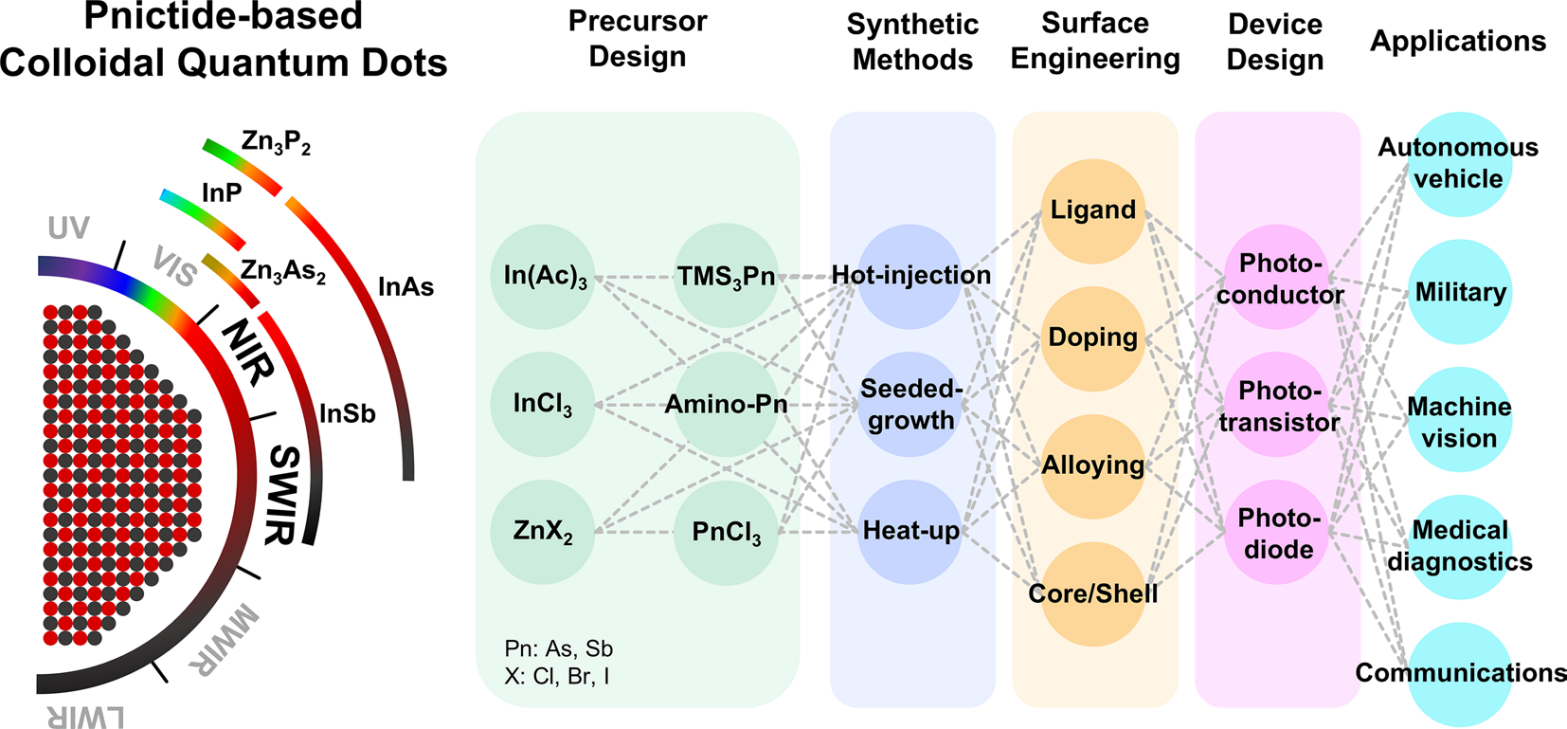
From precursor selection to applications, an overview of pnictide-based colloidal QDs and their integration into advanced optoelectronic devices

In response to these challenges, pnictide QDs (II–V and III–V) have emerged as promising heavy-metal-free alternatives. In particular, III–V QDs such as InP, InAs, and InSb offer advantages over conventional QDs due to their small carrier effective masses, direct bandgaps, and superior optoelectronic properties [32–35]. While InP has been widely utilized in QD-based display technologies [36–38], InAs and InSb QDs have recently demonstrated remarkable potential for SWIR photodetection (Fig. 1) [39, 40]. Recent advances in the synthesis, characterization, and device-level integration of III–V QDs have led to significant improvements in performance. However, challenges remain in precise size control, surface passivation, and ligand engineering to fully optimize these materials for next-generation optoelectronic devices. For SWIR applications, increasing the QD size is essential to extend absorption deeper into the infrared region while preserving favorable electronic properties [41, 42]. Furthermore, the high surface-to-volume ratio and oxophilic nature of III–V QDs make them susceptible to oxidation and defect formation, which can degrade device performance [43–45]. 

To overcome these obstacles, significant research efforts have been directed toward developing advanced passivation techniques, ligand exchange strategies, and doping methods that enhance the optical and electrical performance of III–V QDs [46–48]. Recent progress in pnictide QD research has not only improved their optoelectronic properties but also expanded the availability of precursor materials, enabling greater control over composition and alloying strategies [49]. This has facilitated the development of ternary and quaternary alloys, as well as II–V pnictide compounds, further broadening the material landscape for SWIR applications [50]. While significant strides have been made in charge injection, transport, and interface engineering, further research is required to refine device stability and ensure consistent performance under real-world conditions. A deeper understanding of degradation mechanisms, interfacial charge dynamics, and encapsulation strategies will be crucial for transitioning III–V QDs from laboratory-scale research to commercial IR sensing technologies. In particular, the application of III–V QDs in infrared focal plane arrays (FPAs) remains largely unexplored. While heavy-metal-based QDs (e.g., PbS and HgTe) have been successfully monolithically integrated with CMOS readout integrated circuits via solution-based processes, similar demonstrations with III–V QDs have not yet been realized [51, 52]. This is primarily due to difficulties achieving film uniformity over large areas, pixel-level patterning precision, and sufficient resistance to oxidation and processing-related degradation. Nevertheless, ongoing progress in scalable synthesis, interface engineering, and encapsulation strategies is gradually addressing these limitations, paving the way for the eventual integration of III–V QDs into high-resolution, next-generation imaging platforms.

In this review, we present an in-depth discussion on recent advancements in colloidal pnictide QDs, emphasizing their potential for IR photodetection. The review is organized into three main sections. First, we examine the latest synthesis strategies employed for III–V QDs, highlighting advancements in precursor design, growth mechanisms, size- and composition-control, and key structural characteristics. Second, we explore the role of surface engineering in modifying optical and electronic properties, including ligand exchange strategies, doping, surface treatment, and the design of core/shell architecture. Third, we analyze the integration of III–V QDs into various IR photodetectors, including photoconductors, phototransistors, and photodiodes, assessing recent breakthroughs in device performance and efficiency. Finally, we conclude with a perspective on the challenges and future directions for advancing pnictide QDs toward highly stable, high performance IR sensing technologies.

## Colloidal synthesis and characterization of III–V QDs

The synthesis of colloidal III–V QDs relies on precursor conversion kinetics, nucleation, and growth processes, which collectively determine their size, shape, and optical properties [53, 54]. Due to the strong covalent bonding nature of III–V nanocrystals, their nucleation and growth mechanisms are more complex compared to II–VI and IV–VI QDs, where precursor dissociation and bond formation occur relatively more readily [55]. Therefore, the choice of precursors and reaction conditions is crucial for synthesizing high-quality III–V QDs [56]. In particular, controlling the reactivity of group V precursors is critical for achieving precise size control and desirable optical properties, as their decomposition kinetics directly influence monomer availability and nucleation behavior [49]. A widely used approach for synthesizing III–V QDs is solution-phase synthesis, which includes the hot injection, heat-up, and seeded-growth methods. To provide a contextual overview for the following discussion, Table 1 summarizes representative examples of III–V QDs synthesized via these solution-phase methods. It outlines key synthetic parameters—including precursor types, reducing agents, and reaction temperatures—as well as the resulting excitonic peak positions that reflect the optical characteristics of the QDs.

**Table 1:** Summary of III–V QDs synthesis methods and key parameters

Materials	Methods	Precursors	Reducing agents	Temperature [℃]	Excition peaks [nm]	Ref
InAs	Hot-injection	InCl_3_, amino-As	Amino-P	190	800	[57]
InAs	Hot-injection	InCl_3_, amino-As	Amino-P	180 − 240	1130 − 1600	[58]
InAs	Hot-injection	InCl_3_, amino-As	DIBAL-H	240 − 290	750 − 1450	[59]
InAs	Hot-injection	InCl_3_, amino-As	DIBAL-H	270	1100 − 1700	[60]
InSb	Hot-injection	Indium tris[bis(trimethylsilyl)amide], amino-Sb	n/a	TOP, 130 − 200 TOA, 200 − 300	1250 − 1750	[61]
InSb	Hot-injection	InCl_3_, amino-Sb	Superhydride	250	n/a	[62]
InAs	Seeded-growth	In-oleate, TMS_3_As	n/a	270 − 300	700 − 1110	[63]
InAs	Seeded-growth	In-oleate, TMS_3_As	n/a	300	900 − 1600	[64]
InAs	Seeded-growth	InCl_3_, amino-As	HMTS	260	850 − 1550	[65]
InSb	Seeded-growth	In-oleate, TMS_3_Sb	n/a	300	n/a	[66]
InAsSb	Heat-up	InCl_3_, AsCl_3_	Superhydride	240 − 320	800 − 1800	[49]
InAs	Heat-up	InCl_3_, AsCl_3_	Superhydride	280 − 340	1000 − 1500	[67]
InAs (nanorod)	Heat-up	InCl_3_, AsI_3_	Superhydride	180 − 220	1200 − 1800	[68]
InSb	Heat-up	InCl_3_, Sb[N(Si(Me)_3_)_2_]_3_	Superhydride	240 − 260	1200 − 1750	[69]
InSb	Heat-up	InCl_3_, AsCl_3_	Superhydride	240	1250 − 1860	[70]
InSb	Heat-up	In(I)X (X = Cl, Br, I), amino-Sb	n/a	230 − 280	630 − 1890	[42]
InSb	Heat-up	InCl_3_, TMS_3_Sb	n/a	240 − 300	990 − 1770	[71]

**Fig. 2:**
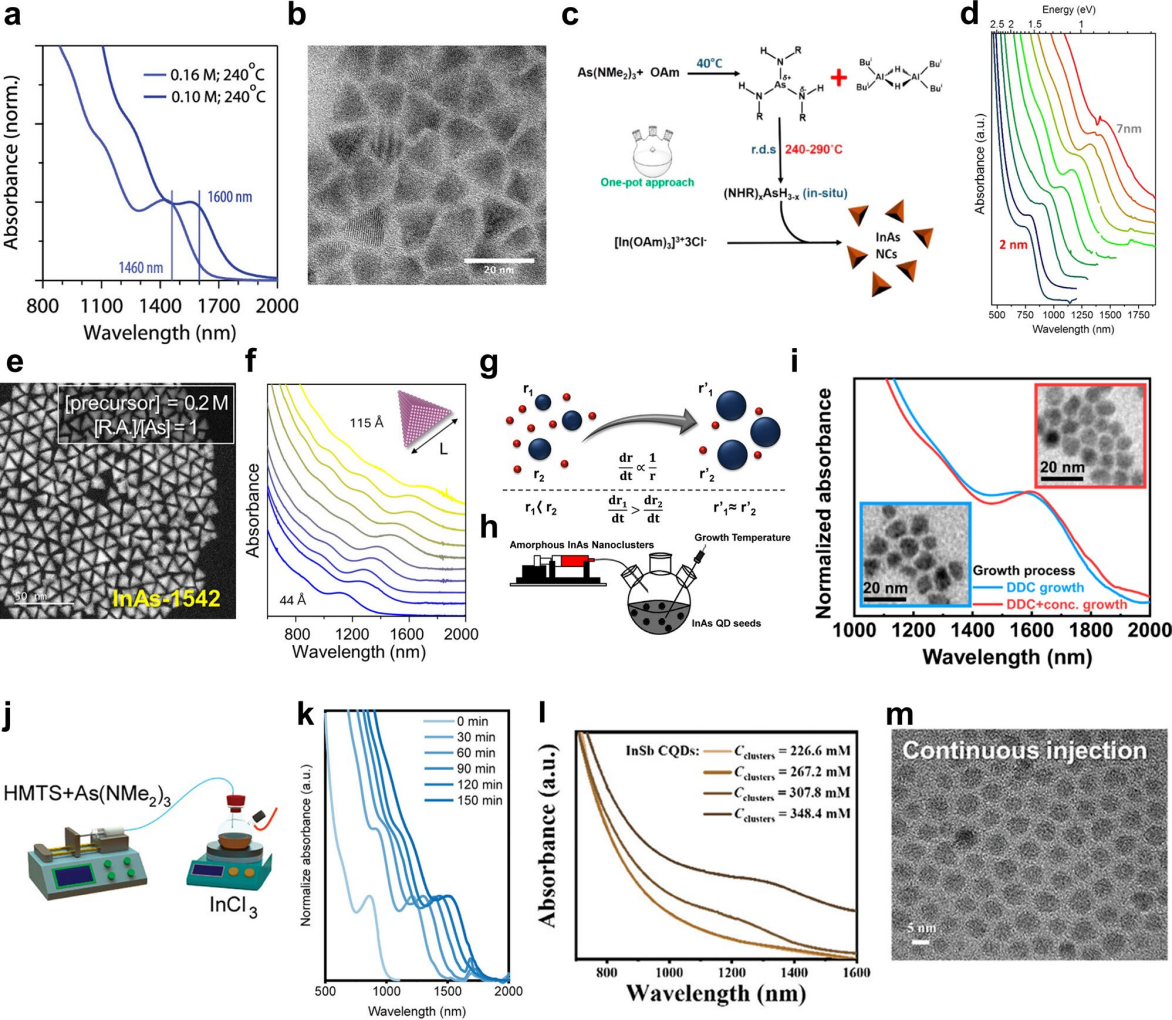
Synthesis of colloidal III–V QDs via the hot-injection and seeded-growth methods. **a** Absorption spectra of the final reaction product for In(As, P) QD syntheses by combining the lowest reaction concentration and the highest injection temperature feasible. **b** TEM images of In(As, P) QDs with a band transition at 1600 nm. Reprinted with permission from [58]. Copyright 2023 American Chemical Society. **c** Scheme for activation of the aminoarsine precursor with DIBAL-H. **d** Absorption spectra of InAs QDs in the size range of ∼2 to ∼7 nm showing tunability of the synthetic protocol. Reprinted with permission from [59]. Copyright 2016 American Chemical Society. **e** HAADF-STEM image of tetrahedral InAs QDs. **f** Absorption spectra of tetrahedral InAs QDs in various sizes of edge length from 4.4 ± 0.6 to 11.6 ± 1.1 nm. Reprinted with permission from [60]. Copyright 2024 American Chemical Society. **g** Illustration of the size-focused growth in which smaller nanocrystals (r_1_) grow faster than larger ones (r_2_), resulting in better size uniformity as the reaction progresses. Only two sized nanocrystals (blue dots) have been shown for simplicity. Red dots symbolize monomers. **h** Synthetic scheme of the growth of InAs QDs via slow and continuous injection of amorphous PNC solution into preformed InAs QD seeds using a syringe pump. Reprinted with permission from [63]. Copyright 2016 American Chemical Society. **i** Absorption spectra of InAs QDs grown using DDC for 9 h showing 1st excitonic peaks at 1579 nm (blue line) and using DDC with additional concentration control for 10 h showing 1600 nm (red line), respectively. Inset: TEM images of InAs QDs. Reprinted with permission from [64]. Copyright 2021 Spring Nature. **j** Seeded-growth reaction scheme for the synthesis of InAs QDs via continuous injection of hydridosilane and amino-As simultaneously. **k** Absorption spectra of aliquots taken from the crude reaction mixture during the reaction using various precursor amounts. Reprinted with permission from [65]. Copyright 2024 John Wiley and Sons. **l** Absorbance of InSb QD as a function of cluster concentration. **m** TEM images of InSb QD synthesized at concentration of 348.4 mm. Reprinted with permission from [66]. Copyright 2024 John Wiley and Sons

### Hot-injection methods

Hot-injection method is widely used approach for various nanocrystals synthesis. Synthesizing InAs using the hot-injection method with tris(trimethylsilyl)arsine (TMS_3_As) was the first approach and the most typical precursor for colloidal InAs synthesis [72]. Injecting TMS_3_As into indium precursor, mainly indium acetate (In(Ac)_3_) and indium chloride, at high temperature induces burst nucleation and leads to formation of uniform particles [73, 74]. However, due to its high reactivity, pyrophoric nature, and handling challenges, TMS_3_As requires careful control of reaction conditions for safe and reproducible synthesis. While band-edge absorption peaks extending beyond 1500 nm have been demonstrated through carefully optimized synthetic conditions, achieving reproducible and uniform growth of such large InAs QDs remains technically demanding—particularly when using highly reactive precursors such as TMS_3_As [63]. 

Tris(dimethylamino)arsine (As(NMe_2_)_3_, amino-As) has been proposed as an alternative due to its commercially availability, safe-to-handle, and lower cost, making it a more practical choice for InAs nanocrystal synthesis [57, 75]. Grigel et al. reported the synthesis of InAs via the hot-injection method using InCl_3_ and amino-As along with tris(diethylamino)phosphine (P(Net_2_)_3_, amino-P) as a reducing agent, thereby avoiding the use of highly reactive or hazardous precursors [57]. In this study, they noted that, unlike the conventional InP synthesis using amino-P, which proceeds without the need for an additional reducing agent, InAs synthesis requires one due to the absence of disproportionation in amino-As. Building upon this approach, Leemans et al. optimized the synthesis to yield QDs with tunable properties suitable for SWIR detection [58]. To achieve uniform and large particles, they decoupled the nucleation and growth processes by initiating nucleation at 190 ℃ and subsequently ramping the temperature to promote controlled growth. Adjusting the reaction temperature, temperature ramp rate, and precursor concentration enabled the synthesis of large, triangular-shaped QDs, exhibiting an absorption peak extending up to 1600 nm (Fig. 2a, b).

Several studies have also reported the hot-injection synthesis using InCl_3_ and amino-As with alternative reducing agents [54]. Srivastava et al. used diisobutylaluminum hydride (DIBAL-H) as a reducing agent, which activated amino-As to form intermediate complex containing As-H bonds that reacts with the indium precursor (Fig. 2c) [59]. Adjusting its concentration and varying the reaction temperature from 240 ℃ to 290 ℃, they achieved QD size control from ~ 2 nm to 7 nm, leading to a shift in the first exciton peak from 750 nm to 1450 nm, while exhibiting a tetrahedral shape (Fig. 2d). Kim et al. further refined this approach by employing lithium bis(trimethylsilyl)amide (LiHMDS) to suppress transamination, thereby slowing the reaction kinetics. The reduction in precursor reactivity slows the buildup of supersaturation, thus suppressing the nucleation burst and favoring sustained growth of existing nuclei [60]. As shown in Fig. 2e, this modification led to the formation of tetrahedral InAs QDs, where the dominance of (111) facets influenced the electronic structure, leading to weakened quantum confinement effect. As a consequence, the first exciton peak extended from 1100 nm to 1700 nm, underscoring their suitability for SWIR applications (Fig. 2f).

In InSb synthesis, tris(trimethylsilyl)antimony (TMS_3_Sb)-based approach has been less commonly employed due to its significantly lower stability, high reactivity compared to TMS_3_As and TMS_3_P, resulting in a broad size distribution and poor crystallinity [76]. Instead, tris(dimethylamino)atimonide (Sb(NMe_2_)_3_, amino-Sb) are more commonly used as Sb precursors for hot-injection method. Yarema et al. explored the synthesis of InSb QDs using amino-Sb with indium tris[bis(trimethylsilyl)amide], demonstrating precise control over polymorphism by adjusting the In/Sb molar ratio, leading to the formation of wurtzite and zinc-blended structures [61]. This approach enabled the synthesis of InSb QDs with tunable optical properties ranging from 1250 nm to 1750 nm. However, In[N(SiMe_3_)_2_]_3_ presents challenges due to its limited commercial availability and complex preparation. Tamang et al. employed in situ activation of InCl_3_ with strong bases such as LiHMDS, n-butyl lithium (nBuLi) and lithium triethylborohydride (superhydride) as a reducing agent [62]. Bases facilitated the formation of reactive species, including In-N and In-C species, while the reducing agent directly reduced InCl_3_ to In(0) species, both contributing to the subsequent reaction with antimony precursor.

### Seeded-growth methods

The conventional hot-injection method poses challenges in size control, which has led to the development of seeded-growth method, employing the continuous injection of precursors to enable the growth of larger particles. This approach effectively separates the nucleation and growth stages, improving monodispersity and allowing for more controlled particle growth [77]. 

Tamang et al. demonstrated the effectiveness of the seeded-growth method by utilizing prenucleation clusters as a monomer source for the growth of InAs QDs [63]. These clusters were synthesized by reacting indium oleate and TMS_3_As at room temperature, forming 1.8 nm-sized amorphous-phase clusters. Separately, InAs seeds were synthesized via a typical hot-injection method, exhibiting a first exciton peak at approximately 1.77 eV. The slow and continuous injection of amorphous InAs clusters into the seed solution promoted further growth of the InAs QDs, increasing their size from approximately 2.5 nm to 5.8 nm (Fig. 2g, h). This process also improved size uniformity, as reflected in the sharpening of the first excitonic absorption peak. The same group further refined the method by introducing a diffusion dynamics-controlled (DDC) synthesis strategy to promote the growth of InAs QDs. Through modeling-based optimization, they systematically regulated monomer flux by tuning key parameters such as reaction volume, precursor concentration, and injection rate. As a result, they achieved large, monodisperse InAs QDs exceeding 9 nm with a first excitonic peak reaching 1600 nm (Fig. 2i) [64]. 

Skorotetcky et al. utilized amino-As and 1,1,3,3,5,5-hexamethyltrisiloxane (HMTS) in the continuous injection method, leveraging the role of Lewis acids in catalyzing the reduction reaction of hydrosilane [65]. By continuously injecting a solution of amino-As and HMTS in oleylamine into a preheated mixture of InCl_3_ and seed solution, they effectively promoted the gradual and controlled growth of QD, where the QD size progressively increased with the precursor injection volume (Fig. 2j). This approach enabled the enlargement of QDs, shifting the first exciton peak from 850 nm to 1550 nm (Fig. 2k). Additionally, it enabled the formation of monodisperse tetrahedral InAs QDs.

Extending its applicability beyond InAs QDs, the continuous injection method was subsequently adapted for the synthesis of InSb QDs, as demonstrated by Jee et al. A cluster solution was prepared by injecting TMS_3_Sb into indium oleate at room temperature, followed by continuous injection into the seed solution at high temperature to synthesized InSb QDs [66]. By adjusting the cluster concentration, both size and dispersity could be controlled. This approach resulted in an exciton transition at 1350 nm, with broadband absorption extending up to 1600 nm (Fig. 2l, m).

### Heat-up methods

**Fig. 3:**
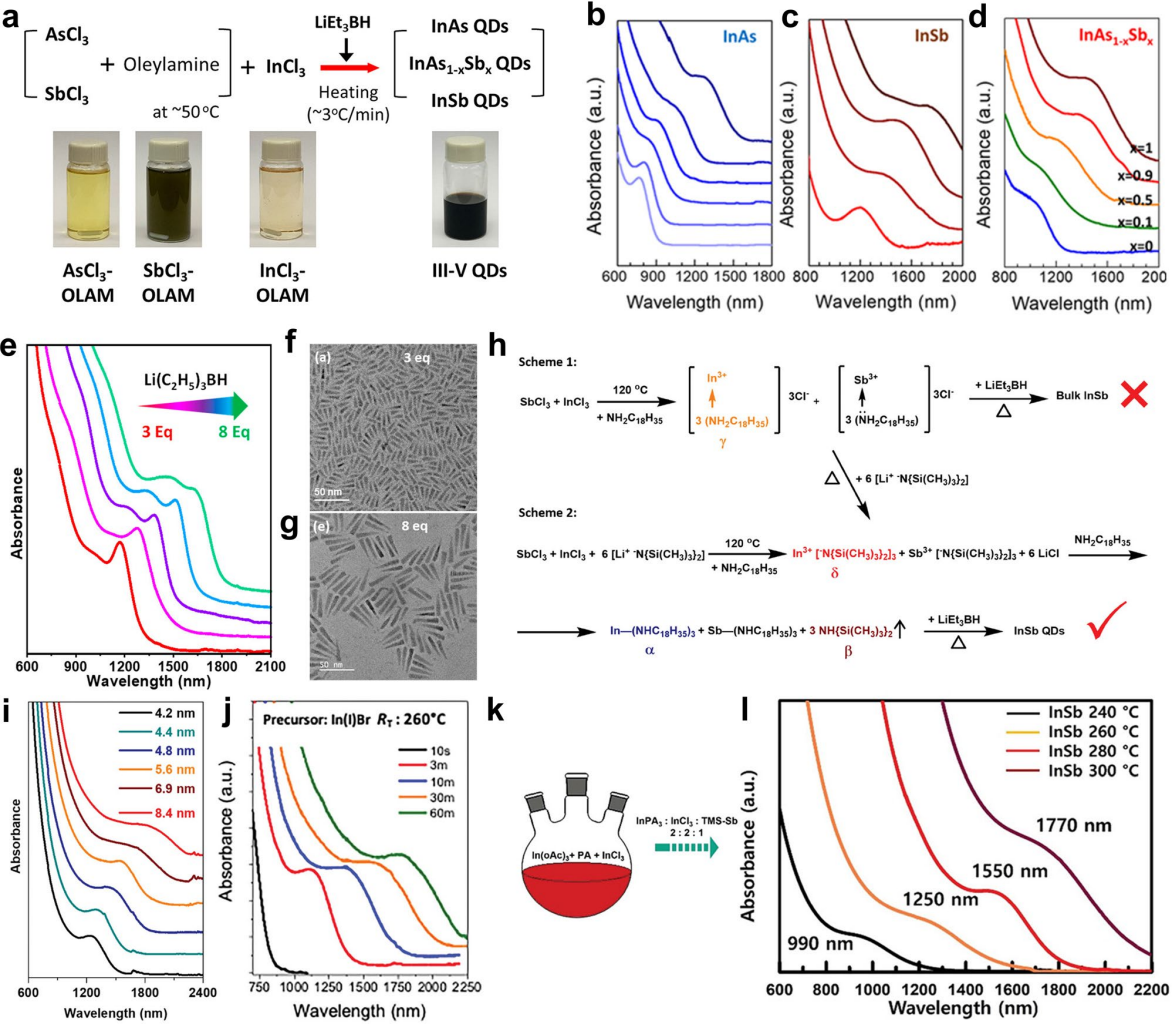
Synthesis of colloidal III–V QDs via the heat-up method. **a** Reaction schematic and photographs of the precursors and product indium-based III–V colloidal QDs. Absorption spectra of **b** InAs QDs synthesized at temperatures of 240 − 340 ℃ in 20 °C steps from light blue to dark blue, **c** InSb QDs synthesized at 260 − 320 ℃ in 20 ℃ steps from red to brown, and **d** InAs_1 − x_Sb_x_ QDs (0 ≤ x ≤ 1) from blue to brown synthesized at 320 ℃. Reprinted with permission from [49]. Copyright 2019 American Chemical Society. **e** Absorption spectra of InAs nanorod QDs synthesized using variable amounts of superhydride. **f**,** g** TEM images of InAs nanorod QDs shown in **e**. Reprinted with permission from [68]. Copyright 2023 American Chemical Society. **h** Scheme 1: Initial reaction scheme for InSb QDs synthesis without LiHMDS. Scheme 2: Optimized reaction scheme for InSb QDs synthesis with LiHMDS addition. **i** Absorption spectra of InSb QDs exhibiting excitonic features in SWIR window ≈ 1250–1860 nm. Reprinted with permission from [70]. Copyright 2023 John Wiley and Sons. **j** Absorption spectra of aliquots taken over time during the syntheses of InSb QDs from In(I)Br at 260 ℃. Reprinted with permission from [42]. Copyright 2024 John Wiley and Sons. **k** Schematic illustration of synthesis with In-oleate, PA, InCl_3_, and TMS_3_Sb. **l** Absorption spectra of InSb QDs depending on growth temperature. Reprinted with permission from [71]. Copyright 2023 John Wiley and Sons

The heat-up method was developed as an alternative to the hot-injection technique to address challenges in scalability and precursor stability for III–V QDs synthesis [78]. In this approach, metal and pnictogen precursors are pre-mixed at room temperature in coordinating solvents and gradually heated, allowing slow reaction without the need for rapid precursor injection. Zhao et al. developed the heat-up method for III–V QDs using InCl_3_ and pnictogen chlorides (PnCl_3_, Pn = As, Sb) as precursors [49]. As shown in Fig. 3a, these precursors were dissolved in oleylamine, forming a stable solution that improved reproducibility and handling. Superhydride, serving as a reducing agent, was then introduced to facilitate the co-reduction of indium and pnictogen precursors, leading to the formation of InAs, InSb and ternary InAs_1 − x_Sb_x_ QDs. The mixture was initially prepared at room temperature, and gradually heated at a rate of 3 ℃/min to the reaction temperature. By adjusting the reaction temperature and precursor ratio, they achieved precise QD size and composition. For InAs QDs, the particle size ranged from 3.0 nm to 6.3 nm as the reaction temperature increased from 240 ℃ to 340 ℃, while InSb QDs varied in size from 4.2 nm to 7.5 nm within the 260 ℃ to 320 ℃ range (Fig. 3b‒d). Ternary InAs_1 − x_Sb_x_ QDs were synthesized by varying the AsCl_3_ and SbCl_3_ precursor ratio, enabling precise control over composition and allowing the absorption region to be tuned from 900 nm to 1800 nm. The final As: Sb ratio closely matched the initial precursor ratio, indicating efficient precursor conversion during synthesis. Expanding upon their previous work, the same group refined the method for InAs QDs synthesis to enhance size control and extend absorption into the SWIR region [67]. The updated approach optimized the [In^3+^]: [As^3+^] ratio and superhydride concentration to achieve larger, tetrahedral InAs QDs, which exhibited absorption extending to 1550 nm.

Conventional InAs QDs are typically spherical and limited in size, restricting their optical tunability below 1400 nm. To address this limitation, Sheikh et al. reported a one-pot synthesis strategy to produce monodisperse InAs nanorod QDs with tunable bandgaps ranging from 1200 to 1800 nm, extending further into the SWIR region (Fig. 3e) [68]. This approach utilized in situ complexation of InCl₃ and AsI₃ with LiHMDS, controlling precursor reactivity to induce anisotropic growth. The formation of indium nanoparticle seeds directed preferential growth along the [111] axis, yielding nanorods with lengths of 15–32.5 nm and widths of 4–6.5 nm (Fig. 3f, g). By tuning the amount of superhydride, size control and narrow excitonic absorption features were achieved, significantly improving optical performance.

Shifting focus to InSb, Liu et al. pioneered the heat-up synthesis of InSb QDs using InCl_3_ and Sb[N(Si(Me)_3_)_2_]_3_ as precursors [69]. The addition of superhydride as a reducing agent generated reactive In(0) and Sb(0) intermediates, initiating the reaction. The mixture was then heated at 3 ℃/min to between 240 ℃ and 260 ℃, yielding nanocrystals with tunable sizes ranging from 3.3 nm to 6.5 nm. The size variation corresponded to bandgaps spanning 1200 nm to 1750 nm with distinctive absorption shoulder.

Wasim et al. refined Zhao et al.’ synthesis method to produce more monodisperse InSb QDs using InCl_3_, SbCl_3_ and superhydride [49, 70]. LiHMDS was introduced during precursor dissolution in oleylamine at 120 ℃, facilitating the in-situ formation of indium- and antimony-silylamide complexes (Fig. 3h). This modification led to balanced reactivity between In and Sb precursors, improving monodispersity and stoichiometric control in the QDs. Superhydride was then injected to initiate the reaction, followed by a gradual temperature increase to 240 ℃. The synthesized InSb QDs exhibited distinct absorption features in the SWIR region of 1250–1860 nm, with size tuning controlled by modulating the reducing agent concentration and employing size-selective precipitation (Fig. 3i).

Kwon et al. synthesized InSb QDs using In(I)X (X = Cl, Br, I) and amino-Sb as precursors [42]. The reaction began with a premixing step at 50 ℃ for 1 h, where the precursors were dissolved in oleylamine to form reactive intermediate. The solution was then heated to 230–280 ℃ at a controlled rate of 20–30 ℃/min, initiating QD nucleation and growth. In(I)X acted as both the indium source and a mild reducing agent, eliminating the need for strong reductants. The method enabled precise size control (2–7 nm), with absorption peaks tunable from 630 nm to 1890 nm, depending on the reaction temperature, reaction time, and halide precursor used (Fig. 3j).

Seo et al. synthesized InSb QDs using a combination of heat-up and hot-injection methods, employing indium oleate, palmitic acid (PA), InCl_3_, TMS_3_Sb as key precursors (Fig. 3k) [71]. The synthesis began with the hot injection of the Sb precursor into a mixture of indium-carboxylate and InCl_3_ solution at 75 ℃, followed by a gradual temperature ramp-up at 3 ℃/min to promote nucleation and growth. By adjusting the final growth temperature from 240 °C to 300 °C, they obtained InSb QDs (2–6 nm) with absorption peaks ranging from 990 to 1770 nm (Fig. 3l). InCl_3_ played a crucial role in modulating precursor reactivity and passivating surface defects, significantly improving the optical properties of the QDs. The presence of Cl⁻ ions from InCl_3_ suppressed Sb oxidation, leading to enhanced surface stability and absorption characteristics. This method enabled precise size control, improved optical properties, and better surface passivation, demonstrating its potential for InSb QD fabrication.

## Surface engineering and characteristic design of III–V QDs

Due to the inherent covalent nature of III–V QDs, they exhibit structural defects that are uncommon in II–VI or IV–VI nanocrystals [79]. Compared to more ionic nanocrystals, surface dangling bonds can introduce trap states that are often positioned deep within the bandgap, posing significant challenges for charge transport and optoelectronic efficiency [32]. Therefore, III–V QDs require more effective passivation strategies to mitigate non-radiative recombination and enhance device performance.

Additionally, because of their strong affinity for oxygen makes III–V QDs highly susceptible to oxidation upon exposure, which modifies surface chemistry and generates electronic defects [80]. This oxidation not only leads to material degradation but also deteriorates interfacial properties. As a result, charge transport is hindered and device reliability is reduced, necessitating additional oxide removal steps [81]. These challenges highlight the importance of precise surface engineering, as both trap state mitigation and oxidation resistance are crucial for optimizing the optoelectronic behavior of III–V QDs.

As a result, advanced surface modification strategies are critical for stabilizing III–V QDs and enhancing their performance in electronic and optoelectronic applications. To address these issues, researchers have focused on improving ligand exchange processes, incorporating doping techniques, developing surface engineering strategies, and designing core-shell structures—all of which contribute to minimizing surface defects, reducing oxidation, and enhancing charge transport properties.

### Ligand exchange

**Fig. 4:**
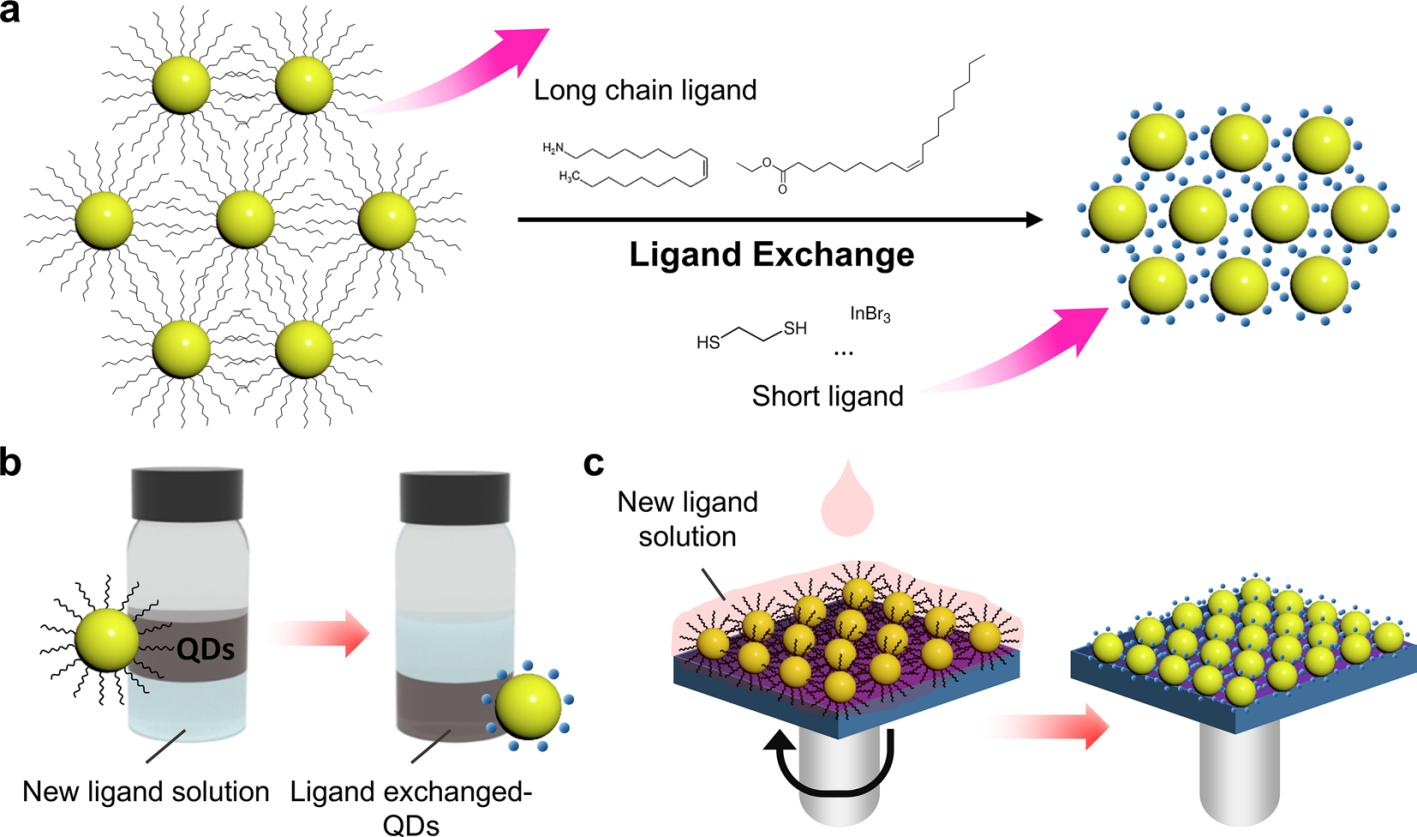
**a** Schematic diagram of the ligand exchange process. The initial QDs capped with long-chain ligands (left) undergo ligand exchange, replacing them with short ligands (right). Schematic illustration of **b** solution-phase ligand exchange and **c** solid-state ligand exchange

Ligand exchange is a process in which the native ligands on the surface of colloidal QDs are replaced with new ligands to tune surface chemistry, improve stability, and promote more efficient electronic coupling between QDs [27, 82]. This step is essential because as-synthesized QDs are typically capped with long-chain insulating organic ligands, which provide colloidal stability but hinder electronic coupling and charge transport in films (Fig. 4a). To integrate QDs into optoelectronic devices, these ligands need to be exchanged with shorter, more conductive ligands, such as halides, chalcogenides, or metal-organic complexes, which facilitate better interdot coupling and carrier mobility.

Solid-state and solution-phase ligand exchange are two primary methods used for ligand engineering. Solution-phase ligand exchange involves dispersing QDs in a nonpolar solvent and introducing a ligand solution in a polar solvent, enabling phase transfer and ligand replacement (Fig. 4b). This method is effective for maintaining colloidal stability and is widely used in ink-based processing for thin-film deposition. In contrast, solid-state ligand exchange is performed directly on pre-deposited films by immersing them in or drop-casting them with ligand solution (Fig. 4c) [83]. This approach enables a controlled reduction in interparticle distance within the film, resulting in a more compact structure that enhances electronic properties. In this work, we introduce various ligand exchange strategies and explore a diverse range of ligands used for quantum dot surface modification, highlighting their influence on electronic properties and surface passivation.

**Fig. 5:**
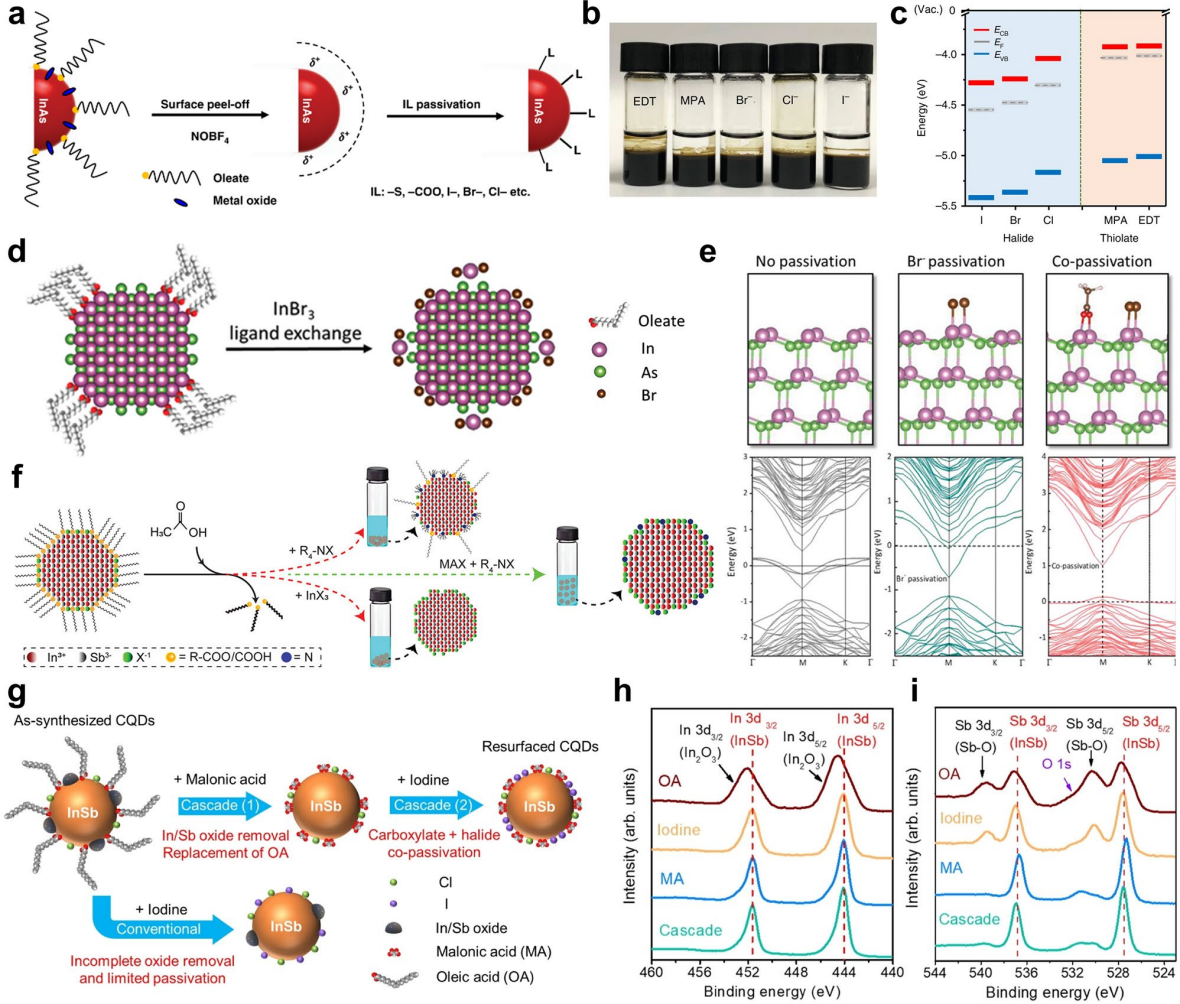
Ligand engineering of colloidal III–V QDs. **a** Scheme of the two-step surface modification of InAs QDs. Step-1 is the clean up of the InAs QD surface by NOBF_4_ treatment; Step-2 is the passivation of a naked surface by incoming ligands (ILs), including thiolate, carboxylate, and halides. **b** Photograph of InAs QDs after surface reconstruction with EDT, MPA, Br, Cl, and I (from left to right). Out of the two phases present, DMF (bottom) and octane (top), surface-reconstructed InAs QDs are dispersed in the DMF. **c** Energy levels of InAs QD films, deduced from UPS measurements. Reprinted with permission from [48]. Copyright 2018 Springer Nature. **d** Scheme of InBr_3_ ligand exchange and InAs QD surface configuration. Reprinted with permission from [46]. Copyright 2022 John Wiley and Sons. **e** Density functional theory (DFT) simulation, side views of ball-and-stick models on InAs (111) facet with naked, pure Br^−^ passivation and co-passivation with Br^−^ and acetate, and their respective band structures near the Fermi energy (dash line). Reprinted with permission from [84]. Copyright 2023 John Wiley and Sons. **f** Scheme of the ligand exchange processes with MAX and TBAX. Reprinted with permission from [85]. Copyright 2023 John Wiley and Sons. **g** Schematic of cascade ligand exchange in comparison with one-step halide exchange. XPS spectra of QDs before and after different surface modifications: **h** In 3d spectra; **i** Sb 3d spectra. Reprinted with permission from [86]. Copyright 2024 John Wiley and Sons

Song et al. employed a two-step ligand exchange process for passivating InAs QDs [48]. Initially, the native oleate ligands and surface oxides were removed using nitrosyl tetrafluoroborate (NOBF_4_), which facilitated ligand stripping and oxide removal through solution-phase transfer. Following this, the QD surfaces were reconstructed with a set of short ligands, including halide (Cl^−^, Br^−^, I^−^) and thiol-based ligands (3-mercaptopropionic acid (MPA) and 1,2-ethanedithiol (EDT)) (Fig. 5a, b). The ligand exchange process resulted in enhanced surface passivation, as confirmed by photoluminescence (PL) analysis, and improved charge transport properties in the film, indicating enhanced electronic coupling between QDs. Additionally, the energy levels of InAs QDs shifted depending on the type of ligand, demonstrating ligand-induced energy level modulation (Fig. 5c). To overcome the limited efficiency of BF_4_^−^ removal in earlier methods, Si et al. introduced an intermediate phase transfer (IPT) strategy to improve ligand replacement [87]. Unlike previous method that directly replaced native long chain ligands, the IPT approach first introduced benzoic acid (BA) to facilitate ligand exchange and transfer the QDs into weakly polar solvents [48, 88]. This preliminary step enabled the effective attachment of thiol based ligand, ensuring more uniform surface passivation.

To address charge imbalance and surface defects, an amphoteric ligand exchange strategy was developed for InAs QDs by Sun et al. [46] Solution phase ligand exchange was employed to introduce InBr_3_ and ammonium acetate (AA), replacing native oleic acid ligands (Fig. 5d). This method simultaneously passivated In and As dangling bonds through X-type (Br^-^) and Z-type (InBr_2_^+^) ligands, effectively neutralizing surface states and reducing trap density. However, this approach still resulted in incomplete surface coverage, limiting colloidal stability and long-term optoelectronic performance. To overcome this limitation, the same research group introduced a sequential co-passivation strategy, incorporating an additional X-type ligand, methylammonium acetate (MaAc), in a second treatment step [84]. This follow-up process step led to enhanced surface coverage, reducing trap-assisted recombination and improving QD stability. The introduction of MaAc resulted in a 25% reduction in Stokes shift, indicating fewer defect states, and doubled the PL lifetime, validating the improved surface quality (Fig. 5e). While this reduction in the Stokes shift is consistent with trap passivation, it is important to note that in III–V QDs, factors such as exciton–phonon coupling and structural disorder can also contribute to the observed spectral features [89]. 

Jung et al. introduced a metal halide ligand exchange strategy through solid-state ligand exchange process with SnX_2_, InX_3_, CdX_2_ (X = Cl, Br, or I) [90]. The efficiency of ligand exchange varied depending on the metal type, with SnX_2_ achieving the highest efficiency, followed by InX_3_ and CdX_2_. Among these, SnBr_2_ exhibited the strongest affinity for As-rich sites, effectively replacing native In-oleate ligand with SnBr^+^ ligands. XPS confirmed the successful removal of In-carboxylate species and the formation of Sn-Br bonds, leading to improved surface coverage and reduced defect states.

For InSb, Muhammad et al. employed a two-phase solution ligand exchange process to replace the native long-chain ligands in InSb QDs with short-halide based ligand [85]. They introduced a mixture of tetrabutylammonium (TBA) and methylammonium (MA) halides, which facilitated ligand exchange (Fig. 5f). Acetic acid was also incorporated, assisting in the removal of native carboxylate ligands and shifting the equilibrium toward halide binding. Seo et al. introduced a cationic molecular metal chalcogenide (MCC) ligands exchange strategy to InSb [71]. Using Mn_2_Se_2_-based MCC ligands, they effectively passivated surface Sb sites, preventing oxidation during device fabrication. Chatterjee et al. applied Sun et al.’ ligand exchange approach to InSb, utilizing InBr_3_ and AA for ligand exchange [46, 91]. Oleylamine-capped InSb QDs were replaced with shorter ligands, leading to a decrease in interparticle distance and an increase in electronic coupling between QDs. Zhang et al. advanced the prior method by developing a cascade ligand exchange approach that improved oxide removal and surface stability [86]. Initially, a dicarboxylic acid (malonic acid, MA) etching step was employed to eliminate native In_2_O_3_ and Sb_2_O_X_ from the QD surface while replacing long-chain oleic acid ligands (Fig. 5g-i). The MA ligands coordinated with surface metal atoms via bidentate bridging mode, further preventing oxidation and reducing interparticle distance. Following this, a halide exchange step using tetrabutylammonium iodide was conducted, leading to co-passivation with carboxylate and halide ligands. A significant reduction in surface oxides and a higher halide-to-In ratio were observed compared to one-step halide exchange.

### Doping and alloying

Doping and alloying are effective strategies for tuning the electronic structure and charge carrier dynamics of QDs, enabling precise control over their electrical and optical properties. Doping allows for modulation of charge carrier concentration by introducing impurity atoms, which can shift the Fermi level, create localized states within the bandgap, and facilitate more efficient carrier flow and injection dynamics [96]. Alloying, on the other hand, enables compositional tuning by incorporating different elements into the host lattice, leading to controlled bandgap engineering and lattice strain modulation. Together, these approaches provide complementary pathways for tailoring the optoelectronic behavior of QDs. Tripathi et al. investigated Cu doping in InAs QDs through a solution-phase reaction, where CuCl_2_ was dissolved in toluene with surfactants and a reducing agent, then mixed with pre-synthesized InAs QDs at room temperature [97]. During doping, Cu atoms diffused into interstitial sites of the nanocrystal lattice, enhancing n-type conductivity. Absorption spectra showed a red-shift at low Cu concentrations due to lattice distortions, while higher doping levels led to a blue-shift, indicating conduction band modulation. Structural analysis confirmed Cu incorporation, and field-effect transistor (FET) measurements showed enhanced mobility and a negative threshold voltage shift, indicating enhanced carrier concentration and conduction.

Asor et al. demonstrated post-synthesis Cd doping to convert intrinsically n-type InAs QDs into p-type, enhancing their electronic properties and stability [98]. The doping process involved dropwise addition of Cd(oleate)₂ to a preheated solution of InAs QDs in 1-octadecene and oleylamine at 260 ℃, ensuring controlled incorporation of Cd impurities. X-ray absorption fine structure (XAFS) analysis revealed substitutional doping, where Cd²⁺ replaced In³⁺ near the QD surface, altering the carrier type. Additionally, X-ray photoelectron spectroscopy (XPS) showed that Cd doping reduced surface oxidation, improving long-term stability. PL measurements exhibited redshift and increased intensity, indicating effective surface passivation. FET characterization further confirmed stable p-type conduction, with Cd acting as both a dopant and a protective layer, mitigating oxidation and enhancing charge transport.

**Fig. 6:**
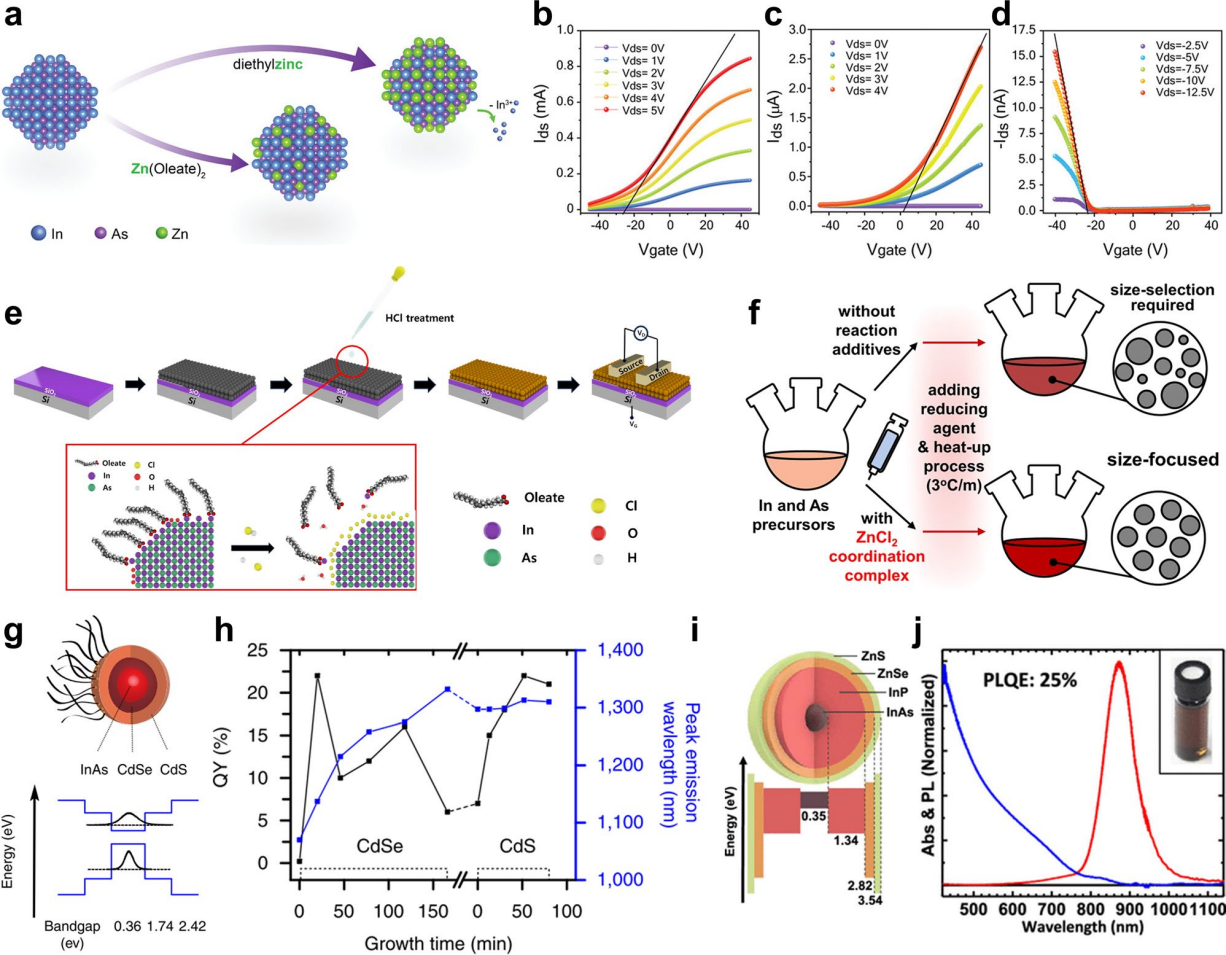
Surface modfication strategies for colloidal III–V QDs. **a** Schematic illustration of the post-synthetic doping reaction with either Zn(oleate)_2_ or diethylzinc. Transfer characteristics of FETs composed of **b** pristine, **c** Zn(oleate)_2_- doped, and **d** diethylzinc-doped InAs QDs indicating the shift in the threshold voltage from heavy n-type to low p-type conduction of the devices. Reprinted with permission from [92]. Copyright 2022 John Wiley and Sons. **e** Scheme of the HCl-treated InAs QD thin-film phototransistor fabrication process. Ligand exchange into the halide and etching of the surface dangling bonds using HCl. Reprinted with permission from [93]. Copyright 2024 Elsevier. **f** Scheme of the colloidal InAs QD syntheses with and without the ZnCl_2_-based coordination complex. Reprinted with permission from [47]. Copyright 2023 Spring Nature. **g** Schematic band alignment in InAs/CdSe/CdS Core-shell-shell QDs. **h** QY and peak emission wavelength evolution during the growth of CdSe and CdS shells on InAs QDs. The CdSe shell causes a redshift in the PL peak and a non-monotonic QY change, indicating a quasi type-II structure. The CdS shell maintains the PL peak position while enhancing QY, suggesting a type-I structure. Reprinted with permission from [94]. Copyright 2016 Spring Nature. **i** Drawing of an InAs − In(Zn)P − ZnSe − ZnS QD with the respective bulk-semiconductor band gaps. **j** Combined absorbance and PL spectra of InAs − In(Zn)P − ZnSe − ZnS QDs. Inset shows an image of the quantum dot solution. Reprinted with permission from [95]. Copyright 2019 American Chemical Society

Building on their previous work, Asor et al. investigated an alternative heavy-metal-free p-type doping approach using Zn dopant. Post-synthetic Zn doping was performed using Zn(oleate)_2_ and diethylzinc, with the latter exhibiting higher reactivity (Fig. 6a) [92]. The dropwise addition of Zn precursors to heated InAs QDs at 260℃ enabled controlled incorporation, where diethylzinc facilitated substitutional doping (Zn^2+^ replacing In^3+^ in the lattice), while Zn(oleate)₂ primarily resulted in surface adsorption as confirmed by XAFS. Zn(oleate)₂-treated QDs exhibited a moderate Fermi level shift, retaining n-type behavior with lower electron mobility, whereas diethylzinc-treated QDs showed a stronger shift, leading to a clear p-type transition. FET measurements confirmed that Zn(oleate)₂-treated QDs remained n-type but exhibited improved conductivity, while diethylzinc-treated QDs achieved p-type conduction with superior charge mobility (Fig. 6b‒d). These results demonstrate that Zn doping effectively modulates carrier type and strengthens carrier flow in InAs QDs. In addition, Yoon et al. reported a synthesis strategy to directly control the polarity of InAs QDs by tailoring the choice of reducing agents [99]. Using amino-As and InCl_3_ as precursors, they achieved p-type InAs QDs by employing diethylzinc as the reducing agent, where Zn atoms serve as substitutional dopants within the InAs lattice. Expanding on the development of Zn-doped InAs, colloidal Zn_3_As_2_ nanocrystals have also been synthesized, exhibiting intrinsic p-type characteristics [50]. These nanocrystals were prepared using a solution-phase approach, and their p-type behavior was confirmed in FET devices.

In addition to doping strategies, compositional alloying offers a powerful route for tailoring the band structure. Srivastava et al. developed a high-temperature cation exchange technique using molten salts (CsBr: LiBr: KBr) as a reaction medium at 380–500 ℃ [100]. In this process, Ga^3+^ partially replaced In^3+^ in preformed InAs QDs, resulting in the formation of ternary In_1 − x_G_x_As QDs. The molten salt environment provides a thermally stable and inert medium, enabling efficient cation exchange at elevated temperature. The resulting QDs exhibited composition-dependent optical properties, with bandgaps tunable from 700 nm to 950 nm as the Ga content increased.

### Surface treatment

The strong covalent nature of III–V QDs makes them highly prone to oxidation and sensitive to surface defects, which significantly impact their electronic properties [80, 101]. Therefore, effective surface modification strategies are required to passivate dangling bonds and suppress defects, enhancing stability and optoelectronic performance. Kim et al. demonstrated that surface fluorination using HF chemically modified the surface of InAs QDs by replacing native long-chain hydrocarbon ligands with fluoride ions [101]. This selective bonding to surface indium sites significantly reduced trap states, resulting in a red shift in absorption and an increase in photoluminescence quantum yield (PLQY). Similarly, HCl treatment has been employed as an effective surface modification strategy for InAs QDs. Choi et al. demonstrated that HCl selectively etches native oxides, such as In₂O₃ and As₂O₃, while protonating surface In sites, which promotes the adsorption of Cl⁻ ligands [93]. This process enhances chemical coverage, reduces interparticle distance, and boosts electronic coupling. The treatment typically involves immersing QDs in an HCl solution, followed by washing and drying steps to ensure complete ligand exchange (Fig. 6e). XPS analysis confirmed the removal of surface oxides, while optical measurements revealed a blue shift in absorption, indicating passivation-induced band structure modification. Electrical characterization showed a significant increase in electron mobility and a reduction in trap density, facilitating more efficient carrier transport. Additionally, HCl-treated QDs exhibited greater air stability, minimizing oxidation-induced degradation. These findings underscore the role of acid-based surface treatment in optimizing the performance of InAs QDs for optoelectronic applications.

Kim et al. utilized a Zn-based coordination complex during the co-reduction synthesis of InAs QDs to enhance surface passivation and oxidation resistance (Fig. 6f) [47]. The in-situ incorporation of ZnCl_2_-trioctylphosphine complex effectively removed surface oxide species and improved colloidal stability. This process also contributed to better size uniformity by modifying the nucleation and growth kinetics, while simultaneously passivating surface defects. XPS confirmed reduced surface oxidation, while optical analysis indicated a higher PLQY due to fewer surface trap states. Electrical characterization demonstrated a shift in energy levels and improved charge transport, highlighting the role of Zn in stabilizing and tuning the electronic properties of InAs QDs.

### Core/shell structure

The formation of core/shell structures has been extensively studied as an effective strategy to mitigate defects in III–V QDs and enhance their overall stability. By encapsulating the core with a protective shell, surface defects that act as non-radiative recombination centers can be passivated, leading to significant improvements in quantum yield, optical stability, and structural durability [12, 102, 103]. Furthermore, depending on the band alignment between the core and shell materials, different heterostructures can be realized, most notably Type-I and Type-II configurations. In a Type-I heterostructure, both electrons and holes are confined within the core, enhancing radiative recombination efficiency and optical properties [104, 105]. This design is beneficial for applications requiring strong PL, such as light-emitting diodes (LEDs) and bioimaging. In contrast, a Type-II heterostructure promotes spatial separation of photogenerated carriers, with electrons and holes predominantly localized in different regions of the core/shell system [106, 107]. This charge separation effect reduces recombination rates and extends carrier lifetimes, making Type-II QDs highly suitable for photovoltaic and photodetector applications.

In an early study, Cao et al. investigated the growth of InP and CdSe shells on InAs QDs to compare their optical properties. Both core/shell structures demonstrated tunable absorption in the infrared region. However, the InAs/CdSe system exhibited a significant enhancement in PLQY, attributed to the minimal lattice mismatch between InAs and CdSe, which facilitated the formation of a highly crystalline shell. In contrast, the InAs/InP core/shell structure follows a Type-I band alignment, where both electrons and holes are confined within the core, but still exhibited reduced PL intensity, highlighting the crucial role of surface quality in determining QY [108]. To improve the QY of InAs QDs for in vivo imaging, Zimmer et al. replaced Cd-based shells with ZnSe, which improved stability and optical properties, making the QDs more suitable for biological applications [109]. However, the significant lattice mismatch (~ 7%) between InAs and ZnSe introduced undesirable strain effects, limiting the efficiency of the core/shell structure. Aharoni et al. introduced a CdSe buffer layer between the InAs core and ZnSe shell to mitigate this issue, effectively reducing strain and enhancing the QY by up to 70% [110]. While the CdSe interlayer improved performance, its small conduction band offset with InAs led to the formation of a quasi type-II band alignment, causing partial electron delocalization into the shell [111]. This effect resulted in extended radiative lifetimes and increased exciton-phonon coupling, both of which negatively impacted QY. To counteract these drawbacks, Franke et al. introduced an additional high-bandgap outer shell of CdS or ZnS, which effectively confined the exciton within the core-shell system, significantly improving QY and further stabilizing the structure (Fig. 6g, h) [94]. To develop heavy-metal-free QDs, the Tan group fabricated InAs QDs with a multi-shell structure comprising InP, ZnSe, and ZnS, effectively passivating surface defects and mitigating lattice mismatch. This approach resulted in a high QY of 25% and enhanced optical stability (Fig. 6i, j). Specifically, they employed a continuous-injection method to grow thick In(Zn)P–ZnSe–ZnS shells around InAs cores, achieving efficient NIR emission with a large Stokes shift and minimized reabsorption losses [95]. Subsequently, the Tan group further optimized the shell structure by introducing GaP as an intermediate shell layer, resulting in In(Zn)As–In(Zn)P–GaP–ZnS core/multi-shell QDs. This design more effectively relaxed the lattice strain and suppressed trap states, leading to a significantly enhanced PLQY of 73% [112]. 

For InSb QDs, Liu et al. enhanced their optical properties by growing CdSe and CdS shells, significantly improving PL efficiency and achieving a remarkable 200-fold increase in PL intensity compared to the original InSb cores [69]. However, CdTe was unsuitable as a shell material because Te tended to form alloys or react with InSb, leading to undesirable byproducts that disrupted the core-shell interface [113]. Seo et al. enhanced the PL and optical stability of InSb QDs by coating them with InAs and ZnSe shells. Since ZnSe and ZnS exhibit a large lattice mismatch when directly grown on InSb, they first introduced an InAs shell to form a Type-I heterostructure, effectively confining both electrons and holes within the core. This approach not only improved PL but also allowed for the subsequent ZnSe shell coating, further enhancing the optical stability of the QDs [71]. Peng et al. synthesized InSb QDs with an InP shell, effectively passivating surface defects and enhancing optical properties. This approach resulted in a PLQY of 3.7% at 1300 nm [114]. 

## III–V QDs-based photodetectors and electrical properties

Photodetectors respond to incident light signals depending on their wavelength. Traditionally, IR photodetectors have relied on absorption layers composed of materials such as PbS, PbSe, PbTe, and HgTe [115–118]. However, with advancements in the size control, synthesis optimization, and surface chemistry engineering of III–V QDs, their application in photon detection has been actively explored.

Infrared photodetector devices can be broadly categorized into three types: photoconductors, phototransistors, and photodiodes. To evaluate and compare the performance of these different types of photodetectors, several key parameters must be considered. Among these, responsivity (*R*) is a critical parameter that quantifies the generated photocurrent (*I*_*ph*_) or photovoltage (*V*_*ph*_) in response to the incident optical power (*P*_*in*_), as described by the following equation:

$$\:{R}_{I}=\:\frac{{I}_{ph}}{{P}_{in}}\:\text{o}\text{r}\:{R}_{v}=\:\frac{{V}_{ph}}{{P}_{in}}$$

Where R is expressed in units of A/W (for photocurrent) or V/W (for photovoltage). A higher responsivity indicates greater sensitivity to incident light, which is essential for efficient photon detection. Another key parameter used to characterize photodetector performance is detectivity (D*), also known as specific detectivity. It enables direct comparison between different devices by normalizing the signal-to-noise characteristics. D* is defined as the reciprocal of the noise-equivalent power (*NEP*), normalized by the square root of the detector`s active area (*A*) and the frequency bandwidth (*∆f*), expressed by:

$$\:{D}^{*}=\frac{\sqrt{A\cdot\:\varDelta\:f}}{NEP}\:\text{o}\text{r}\:{D}^{*}=R\cdot\:\frac{\sqrt{A\cdot\:\varDelta\:f}}{{i}_{n}}$$

Since NEP is related to *R* and noise current density (*i*_n_) as:

$$\:NEP=\:\frac{{i}_{n}}{R}$$

where *i*_*n*_ is the noise current density (A/Hz^1/2^), *R* is the responsivity (A/W or V/W), *A* is the effective detector area (cm^2^), and *∆f* is the bandwidth (Hz). NEP is a fundamental parameter that defines the minimum detectable optical power of a photodetector. It represents the optical power at which the signal-to-noise ratio (SNR) equals 1 under a 1 Hz bandwidth condition. Since D* normalizes NEP by accounting for the detector area and bandwidth, it provides a standardized measure of sensitivity. Higher D* values indicate superior performance, making it a crucial figure of merit for high-performance IR photodetectors. D* is expressed in units of Jones (cm∙Hz^1/2^/W). Another important performance metric is external quantum efficiency (EQE), which refers to the ratio between the number of charge carriers generated as photocurrent to the number of incident photons on the photosensitive materials. It provides insight into how efficiently a photodetector converts incoming photons into electrical signals. EQE is mathematically related to *R* and is expressed as:

$$\:EQE=\:\frac{R\cdot\:hc}{q\lambda\:}$$

*q* is the elementary charge of an electron (1.602 × 10^− 19^ C), *h* is Plank`s constant (6.626 × 10^− 34^ J∙s), *c* is the speed of light (3.0 × 10^8^ m/s), λ is the wavelength of the incident light (m), and R is the responsivity (A/W).

In this section, we will provide an overview of various photodetector types, with a particular focus on photodiodes, which are widely used for their fast response time and high sensitivity. However, photoconductors and phototransistors also play important roles in IR detection, offering distinct advantages depending on the application. The following sections will explore the characteristics and performance of these different types of photodetectors, comparing their key figures of merit such as *R*, D*, and EQE. Table 2 summarizes the electrical properties and device structure discussed in this review.

**Table 2:** Summary of recent III–V QD-based SWIR photodetector performances

Material	Device structure	Responsivity [A/W] @Wavelength (nm) @ bias [V]	EQE (%) @Wavelength (nm) @ bias [V]	Detectivity (Jones) @Wavelength (nm) @ bias [V]	Ref
InAs	Photoconductor	n/a	0.8% @ 1350 nm	n/a	[49]
InSb	Photoconductor	0.4 × 10^− 3^ @ 1400 nm	n/a	1.5 × 10^6^ @ 1400 nm	[119]
InAs	Phototransistor	1.15 × 10^5^ @ 905 nm @ -7 V (V_G_)	3.72 × 10^6^ @ 905 nm @ -7 V (V_G_)	5.32 × 10^16^ @ 905 nm @ -7 V (V_G_)	[120]
InAs	Phototransistor	1.134 @ 1060 nm	n/a	2.18 × 10^10^ @ 1060 nm @ 30 V (V_G_)	[121]
InAs	Photodiode	5 @ 1050 nm @ -2 V	n/a	2.98 ± 0.46 × 10^8^ @ 1050 nm	[47]
InAs	Photodiode	0.22 @ 940 nm @ 0 V	30% @ 940 nm @ 0 V	10^11^ @ 940 nm	[46]
InAs	Photodiode	n/a	37% @ 950 nm @ 0 V	1.9 × 10^11^ @ 950 nm	[84]
InAs	Photodiode	0.6 @ 940 nm @ -3 V	79% @ 940 nm @ -3 V	3.1 × 10^11^ @ 940 nm @ -1 V	[122]
InAs (nanorod)	Photodiode	0.17 @ 1450 nm @ -1 V	14.9% @ 1450 nm @ -1 V	1.2 × 10^10^ @ 1450 nm @ -1 V	[123]
InAs	Photodiode	n/a	28% @ 950 nm @ 1 V	4 × 10^11^ @ 950 nm @ 1 V	[124]
InAs	Photodiode	n/a	36% @ 940 nm @ -1 V	1.6 × 10^11^ @ 940 nm	[88]
InAsP	Photodiode	0.007 @ 1400 nm	0.9% @ 1400 nm @ -4 V	1.0 × 10^9^ @ 1400 nm	[125]
InSb	Photodiode	0.078 @ 1520 nm @ 0 V	6.3% @ 1520 nm @ 0 V	3.6 × 10^12^ @ 1520 nm @ 0 V	[71]
InSb/InP (Core/Shell)	Photodiode	n/a	25% @ 1240 nm @ 2 V	4.4 × 10^11^ @ 1240 nm @ 0 V	[114]
InSb	Photodiode	n/a	74.4% @ 1200 nm @ 1 V	1.02 × 10^11^ @ 1200 nm	[85]
InSb	Photodiode	0.098 @ 1200 nm @ -1 V	10.1% @ 1200 nm @ -1 V	n/a	[91]
InSb	Photodiode	0.28 @ 1400 nm	25% @ 1400 nm @ 1 V	1.4 × 10^11^ @ 1400 nm	[86]
InSb: InAs	Photodiode	3.76 × 10^− 3^ @ 1550 nm	n/a	2.7 × 10^8^ @ 1550 nm	[66]

**Fig. 7:**
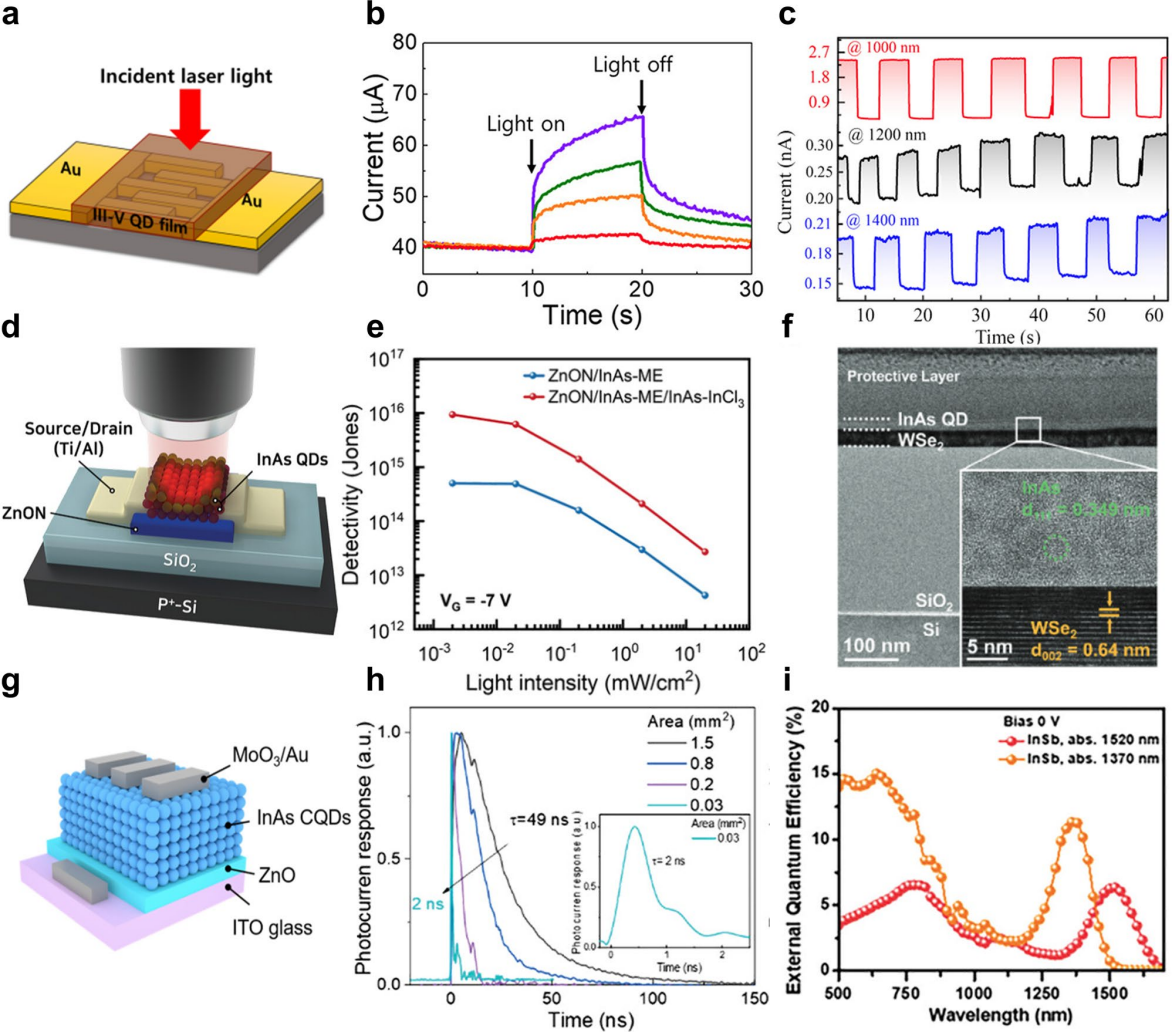
Three types of photodetectors and their electrical characteristics. **a** Schematic illustration of a photoconductor using III–V QDs. **b** Photoresponse of InAs QD photoconductors illuminated at 530 (purple), 650 (green), 1100 (orange), and 1350 (red) nm. Reprinted with permission from [49]. Copyright 2019 by American Chemical Society. **c** Photoresponse of InSb QD photoconductors under illumination at 1000, 1200, and 1400 nm. Reprinted with permission from [119]. Copyright 2022 by John Wiley and Sons. **d** Schematic illustraion of an InAs/ZnON phototransistor. **e** Detectivity of an InAs QD-based phototransistor as a function of optical light power densities. Reprinted with permission from [120]. Copyright 2023 by John Wiley and Sons. **f** cross-sectional TEM imgae of WSe_2_/InAs heterojunction device. Reprinted with permission from [121]. Copyright 2025 by John Wiley and Sons. **g** Schematic illustraion of a photodiode using InAs QDs. Reprinted with permission from [88]. Copyright 2021 by American Chmical Society. **h** Response time of InAs QD photodiodes. Reprinted with permission from [46]. Copyright 2022 by John Wiley and Sons. **i** Device performance of InSb-basd photodiode in terms of EQE. Reprinted with permission from [71]. Copyright 2023 by John Wiley and Sons

### Photoconductors

A photoconductor is a two-terminal device and one of the simplest types of photodetectors, consisting of two electrodes and a photoactive layer (e.g., QDs) (Fig. 7a). Its operation is based on a change in carrier density or mobility when the photoactive layer is exposed to incident light under an applied bias. When an electric field is applied, majority carriers move through the photoactive layer faster than minority carriers due to their higher mobility. As a result, once the majority carriers have traversed the layer, the electrodes can inject additional carriers to maintain charge neutrality, leading to the generation of secondary photocurrent. This process enhances the overall photocurrent response of the photoconductor.

Zhao et al. employed a hybrid ligand exchange to optimize InAs QDs for photoconductor [49]. This approach involved solution-phase ligand exchange using Na_2_S and solid-state ligand exchange using NaN_3_. As a result, the EQE reached at 3.6% at 1100 nm and 0.8% at 1350 nm, demonstrating the effectiveness of the hybrid ligand exchange process in enhancing infrared photoresponse (Fig. 7b). He et al. incorporated InSb QDs with PCBM and poly-TPD to improve the junction quality with the metal contact and achieve better energy alignment. As a result, the device achieved a *R* of 0.04 mA/W at 1400 nm and a D* of 1.5 × 10^6^ Jones (Fig. 7c) [119]. 

Photoconductors can achieve high photoconductive gain and high responsivity by applying an external voltage. However, they suffer from high noise levels, which lead to lower detectivity, and their response time is relatively long. For instance, He et al. reported a response time of 80 ms, indicating one of the key limitations of photoconductors in fast-switching applications.

### Phototransistors

Phototransistors are three-terminal optoelectronic devices that incorporate a gate-controlled semiconducting channel, enabling modulation of charge transport and internal gain. Compared to photoconductors, phototransistors offer lower dark current and higher photoresponse due to the gate-induced control over carrier density. The gain in phototransistors is directly related to carrier mobility, leading to the integration of QD films with high-mobility materials such as transition metal dichalcogenides and amorphous metal oxide semiconductors (Fig. 7d). In this structure, photo-generated carriers from the QD layer are transferred to the high-mobility material and extracted through the source and drain electrodes under V_DS_ bias.

To enhance the performance of QD-based phototransistors, various carrier transport layers have been explored. Kim et al. utilized ZnON (zinc oxynitride), an amorphous metal oxide semiconductor, as the carrier transport layer, while employing InAs QDs as the IR-absorbing layer. To achieve a well-aligned band structure, a graded layer was formed by treating InAs QDs with mercaptoethanol and InCl_3_, leading to a responsivity of 1.15 × 10^5^ A/W and a D* of 5.32 × 10^16^ Jones under 905 nm illumination (Fig. 7e) [120]. Building upon these materials advancements, recent studies have extended the application of QD-base phototransistors beyond conventional photodetection, particularly in neuromorphic systems, where phototransistors can emulate synaptic behavior for artificial sensory processing. Shim et al. fabricated a phototransistor using WSe_2_ as the carrier transport layer and InAs QDs as the absorption layer (Fig. 7f). This device architecture facilitated the observation of synaptic behavior, demonstrating its potential for applications in mimicking biological sensory functions [121]. 

However, despite their advantages, phototransistors still exhibit relatively slow response times and high operating voltages due to large electrode spacing, similar to photoconductors. To overcome these challenges, further optimization of channel materials, interfacial engineering, and device architecture is required to enhance their performance for high-speed and energy-efficient IR photodetection.

### Photodiodes

Photodiodes are vertical optoelectronic devices that exhibit rectification effects and are primarily composed of an electron transport layer (ETL), a hole transport layer (HTL), and a photoactive layer. Their operation relies on the presence of a built-in electric field, which facilitates charge separation and carrier collection, a process often enhanced by the application of reverse bias to improve charge extraction efficiency. Until recently, research on QD-based photodiodes has primarily focused on heavy-metal- based QDs, such as PbS and HgTe QDs, due to their facile synthesis, tunable bandgaps, and well-established surface chemistry [52, 126–128]. In contrast, the development of III–V QDs for photodiode applications has been hindered by synthetic challenges, as mentioned earlier. However, recent advancements in III–V QD synthesis, including precursor engineering, synthetic optimization, and surface chemistry tuning, have enabled the integration of III–V QD based photodiodes.

Choi et al. fabricated a photodiode with the structure ITO/ZnO/InAs QDs/MoO_3_/Au, utilizing InAs QDs as the active layer (Fig. 7g). Their study revealed that indium-carboxylate ligands, which passivate as-synthesized InAs QDs and contribute to an In-rich surface, can be replaced with anionic ligands such as thiols to enhance charge transport. As a result, the device achieved an EQE of 36% at 940 nm and a photoresponse time of 65 ns [88]. Building on this, Sun et al. fabricated a similar ITO/ZnO/InAs QDs/MoO_3_/Au photodiode but further optimized carrier mobility through InBr_3_ treatment, followed by ligand exchange with short ligand to improve charge transport and surface passivation [46]. As a result, the device achieved a *R* of 0.22 A/W, D* of 10^11^ Jones, and EQE of 30% at 940 nm. Furthermore, the photodiode exhibited a -3 dB cutoff frequency of 150 MHz, demonstrating its potential for high-speed IR photodetection (Fig. 7h). To further enhance III–V QD photodiode performance, Xia et al. developed a sequential co-passivation strategy by combining InBr_3_ and methyl ammonium acetate (MaAc). While InBr_3_ ligand exchange enhances carrier mobility, it does not fully passivate the QD surface, leaving unpassivated sites that degrade device performance. By introducing MaAc in a subsequent passivation step, the remaining surface defects were mitigated, leading to improved charge transport and enhanced device stability [84]. In InSb QDs, Zhang et al. mitigated surface oxidation by implementing a dicarboxylic acid-assisted passivation strategy. Malonic acid was used to remove surface oxides, followed by tetrabutylammonium iodide ligand exchange, which achieved co-passivation of the In-rich surface. This approach effectively reduced trap states, leading to an EQE of 25% at 1400 nm and D* of 1.4 × 10^11^ Jones [86]. Furthermore, Ban et al. demonstrated that precursor selection significantly influences the surface composition of InAs QDs. They synthesized InAs QDs using either TMS_3_As or tris(trimetyhlgermyl)arsnine (TMGe_3_As), with TMGe_3_As-derived QDs exhibiting In-rich surfaces prone to trap formation. By applying an InBr₃-based resurfacing protocol, they equalized surface stoichiometry across different synthetic routes, resulting in comparable photodiode performance with an EQE of 28% at 940 nm [124]. 

III–V QDs exhibit strong covalent bonding characteristics, making defect passivation a critical factor in optimizing their optoelectronic properties. Additionally, these QDs suffer from oxidation, which can further degrade device performance [32]. To address these challenges, Zinc halide-based surface treatments have been explored to remove surface traps and improve QD stability effectively [129]. Kim et al. introduced a ZnCl₂:TOP coordination complex during the coreduction synthesis of InAs QDs, which led to improved size uniformity and reduced surface defects [47]. Devices based on Zn-passivated InAs QDs exhibited two orders of magnitude lower dark current and approximately twice the photoresponse speed compared to those using bare InAs QDs. In the case of InSb QDs, Muhammad et al. demonstrated that ZnBr₂ addition effectively improved QD size uniformity. Furthermore, a ligand exchange strategy utilizing tetrabutylammonium and methylammonium halides, along with acetic acid, enabled the complete displacement of native long-chain organic ligands. This process significantly enhanced the performance of InSb QD-based photodiodes, achieving an EQE of 74.4% at 1200 nm [85]. 

Furthermore, efforts to passivate trap states in III–V QDs have included core/shell strategies, where a protective shell is grown around the QD core to enhance stability and reduce surface defects. Sheikh et al. demonstrated that forming a ZnSe shell around InAs nanorods effectively prevents oxidation of the InAs core, leading to improved device performance. As a result, their photodetector exhibited significantly reduced dark current and achieved an EQE of 15% at 1550 nm with a D* of 1.2 × 10^10^ Jones [123]. Peng et al. developed an InSb/InP core-shell structure to mitigate surface defects and enhance photodetector performance. The InP shell effectively passivated trap states, reduced interface dangling bonds, and suppressed Sb oxidation. Furthermore, TiO₂ was employed as ETL instead of ZnO due to its superior photochemical stability. As a result, the photodetector achieved an EQE of 25% at 1240 nm, a wide linear dynamic range exceeding 128 dB, and a fast response time of 70 ns [114]. Seo et al. further advanced the core/shell approach by developing an InSb/InAs/ZnSe core/shell/shell structure. The InAs intermediate shell was introduced to reduce lattice mismatch while the ZnSe outer shell enhanced optical stability and antioxidation properties. This structure improved the photostability of InSb QDs and was applied in photodetector fabrication. As a result, the InSb core photodiode exhibited an EQE of 6.3% at 1520 nm, while the InSb/InAs-based photodiode achieved an EQE of 4.6% at the same wavelength (Fig. 7i) [71]. 

Beyond optimizing the absorber layer, the overall device architecture plays a critical role in determining the performance of QD-based photodiodes. Proper energy level alignment between interlayers is essential to facilitate efficient charge transport, minimize carrier recombination, and suppress leakage currents, all of which contribute to enhanced device efficiency. Leemans et al. demonstrated that a well-matched energy band structure is crucial for improving photodiode performance. They fabricated In(As, P) QD photodiodes by integrating NiO as the hole transport layer and Nb: TiO_2_ as the electron transport layer, achieving a rectifying junction with internal quantum efficiencies of up to 46% at 1400 nm [125]. Similarly, Jee et al. employed a bulk heterojunction (BHJ) approach using n-type InAs and p-type InSb QDs, which resulted in a sixfold enhancement in responsivity compared to conventional single-layer QD devices due to more efficient charge separation and transport [66]. Moreover, Chatterjee et al. emphasized the importance of reducing carrier leakage by optimizing the interface between QD layers and transport layers through the incorporation of PEDOT: PSS and ZnO [91]. 

The thickness of the QD absorber layer plays a crucial role in optimizing the performance of QD-based photodiodes for IR detection. Shin et al. systematically studied the impact of InAs QD layer thickness on device performance, demonstrating that increasing the absorber layer thickness enhances photon absorption, suppresses noise, and reduces resistor-capacitor time, leading to improved photodetector performance [122]. By optimizing the QD layer thickness, their device achieved an EQE of 79%, a *R* of 0.6 A/W, and D* of 3.1 × 10^11^ Jones at 940 nm, with response time of 46 ns.

Photodiodes offer several advantages over other types of IR photodetectors, making them highly suitable for high-performance applications. Their built-in electric field enables efficient charge separation and collection, reducing carrier recombination and enhancing signal-to-noise ratio. Unlike photoconductors, photodiodes typically exhibit lower dark current and faster response times, making them ideal for high-speed and low-noise infrared detection. Furthermore, by carefully engineering the absorbing layer, transport layers, and energy level alignment, recent advancements have significantly improved their D*, EQE, and operational stability. These advantages position QD-based photodiodes as promising candidates for next-generation IR sensing and imaging technologies.

However, translating these device-level advantages into large-area imaging platforms such as FPAs presents additional challenges. The deployment of III–V colloidal QDs in FPAs is still constrained by practical limitations such as achieving spatially uniform film thickness and composition, defining pixel-scale resolution patterns, and preserving material integrity throughout the device fabrication process. In contrast, heavy-metal-based QDs have reached a higher level of technological maturity in this context, supported by well-established ligand chemistries and integration processes. Further progress in scalable deposition methods, fine-patterning techniques, and encapsulation strategies will be necessary to bridge the gap toward FPA-level implementation of III–V QD-based photodiodes.

## Conclusions

In this review, we have outlined the progress in the synthesis, surface engineering, and photodetector integration of infrared III–V QDs, particularly InAs and InSb QDs. Through precursor-driven synthesis optimization, ligand passivation strategies, and heterostructure engineering, significant advancements have been achieved in electronic properties, optical stability, and device integration. These developments have led to improvements in SWIR absorption, charge transport, and overall photodetector performance.

Despite these advancements, challenges remain. III–V QDs still exhibit limitations in SWIR absorption, particularly beyond 1500 nm, and device performance remains suboptimal compared to conventional infrared materials. To overcome these hurdles, further refinements in synthesis techniques are needed to achieve precise size control and enhanced monodispersity, enabling absorption deep into the SWIR region. Additionally, innovative surface passivation strategies must be developed to mitigate surface defects and suppress recombination. Ligand exchange processes should be further optimized to reduce interparticle distance, enhance carrier mobility and improve film conductivity.

Exploring alternative shell materials, doping strategies, and advanced device architectures will be crucial in maximizing photodetector efficiency and stability. Looking ahead, we believe that the next stage of progress in III–V QD-based infrared photodetectors will hinge on the strategic integration of surface engineering, doping, and device architecture. For surface passivation, multi-shell structures—such as those combining InP and ZnSe or ZnS—are promising due to their ability to reduce lattice mismatch, suppress interfacial strain, and enhance carrier mobility, ultimately lowering dark current and improving device stability. Doping strategies such as the incorporation of Zn, Ga, and Cu have shown effectiveness in reducing trap density and tuning band alignment, while emerging dopants like Ag offer additional opportunities to modulate carrier concentration and optical absorption strength. On the device level, PIN-type photodiodes remain a robust architecture for efficient carrier separation and noise suppression. Moreover, integrating III–V QDs with two-dimensional materials such as MoS_2_ or graphene is expected to accelerate charge transfer dynamics and enable flexible, monolithically integrable platforms for infrared sensing. We anticipate that these combined approaches, guided by precise synthetic control and device-materials co-optimization, will be key to advancing III–V QDs toward competitive and scalable SWIR photodetector technologies.

Future research should prioritize precursor engineering and reaction kinetics control to improve size tunability, reproducibility, and scalability of III–V QDs. Additionally, integrating III–V QDs into next-generation photodetectors will require further advancements in charge transport engineering, encapsulation techniques, and device processing strategies. In particular, the development of pixel-level device processing is essential for achieving high-resolution IR imaging cameras, ensuring precise patterning and uniformity in large-area QD films. By addressing these challenges, III–V QDs have the potential to revolutionize QD-based SWIR photodetectors, high-resolution IR imaging systems, wearable night vision devices, and optoelectronic platforms for biomedical and environmental sensing. These advancements will position III–V QDs as key materials in the future of high-performance infrared technologies, bridging the gap between scalable colloidal synthesis and high-efficiency optoelectronic applications.

## Data Availability

Data sharing is not applicable to this article as no datasets were generated or analysed during the current study (review article).
